# On the Heredity of Retinitis Pigmentosa

**Published:** 1875-04

**Authors:** Herman Schmidt


VOL. V.—APRIL, 1875.—No. 4.

### Original Communications.

### ON THE HEREDITY OF RETINITIS PIGMENTOSA.

BY DR. HERMAN SCHMIDT.

[*Translated from the Monatsblätter für Augenheilkunde, by* SWAN M. BURNETT.]

The frequent occurrence of retinitis pigmentosa in a number of children of the same family, makes the existence of an hereditary predisposition probable; and it is worth while to investigate the immediate cause for it. In a certain per cent. of cases, a relationship of the parents can be shown. In order, however, to show that retinitis pigmentosa arises by preference from these marriages, statistics are first needed, which shall give us the exact proportion between the marriages of relatives and those who are not relatives. But still, even when we have this information, we shall be in possession of no definite idea as to the origin of the disease itself.—Only when we find in parents anomalies, whose further development and change can be determined with a degree of certainty, as the pigmentary degeneration of the retina—only then does this phenomenon fall in the well-known and recognized category of transmission of certain peculiarities from parent to offspring.

But even here it is not always the identical affection which is reproduced in the children ; the characters and peculiarities being at one time less marked and retrogressive, while at another they are extended and enlarged in such a degree that only by close observation—but then unmistakably—does the connection with the inherited disposition become apparent.

Not only the bodily appearance, as we all know, but also the intellectual character, give authentic proof of it. Simple eccentricities—a certain neurotic diathesis—can develop itself, as psychologists teach, in succeeding generations into very marked diseases of the mind, and the supposition appears justified, that lesions, which were purely functional in ancestors, can, in course of time, through successive generations, assume an organic form.

From this point of view, a family which presented itself during the past Summer session at the Marburg clinic, possesses some interest.

The father—a peasant, 55 years of age—has seen badly with the right eye, according to his own statement, since childhood. This eye is completely amaurotic, with the exception of a very small portion in the outer quadrant of the visual field, where fingers can be counted on the closest approximation to the eye. Ophthalmoscopic examination reveals an atrophy of the optic papilla. The principal central part of the nerve is excavated and perfectly white. The peripheral ring is of a gray color, and is somewhat broader toward the macula lutea than toward the nasal side. Arteries moderately contracted. The eye squints strongly inward. The left eye is normal.

Examination showed the eyes of the mother to be perfectly sound. By-the-way, it may be remarked that neither she nor her husband were found, upon the closest examination, to be syphilitic.

The oldest daughter, 27 years of age, has a strabismus concomitans convergens, together with occasional nystagmic oscillation. She is emmetropic on both sides, (without atropine), and has normal vision, and a perfect visual field; no hemeralopia. The papilla, however, presents a most peculiar and uncommon appearance, especially as regards color. It is of a pronounced gray, with extremely small white points, presenting a speckled appearance, similar to those cases where, in deep excavation, the lamina cribrosa shines through. It is distinguished from this, however, by a dullness and lustrelessness of color. At the same time the edge of the papilla shades off imperceptibly. The vessels are more than commonly contracted; their origins not abnormally deep. Otherwise the fundus of the eye is normal. Both the other children—a girl, 21 years of age, and a lad of 17 years—suffered with typical retinitis pigmentosa, with alteration in color of the papilla, narrowing of the vessels, and extensive pigmentary deposits. In both, the disease had made most progress in the right eye. The subjective symptoms, hemeralopia and sharpness of vision, began to show themselves in childhood. In the girl, nystagmus and strabismus convergens are present, together with a high degree of hyperopia in both eyes.

This family furnishes us a direct insight into the mysteries of heredity. The father had (possibly congenital?) atrophy of the optic nerve; the oldest child an anomalous conformation of both optic nerves, which caused no functional disturbance: in the youngest children, on the other hand, a typical pigmentary degeneration of the retina had developed itself. The intimate connection between the last and optic-nerve lesion is sufficiently proven, both by clinical and experimental observation.

In connection with this, I will add that in both cases I have obtained a moderate but very constant improvement of the central acuteness of vision by means of the hypodermic injection of strychnia. On the 19th of May, 1873, the girl could only count fingers at one foot with the left eye, and at four inches with the right eye; on the 21st of June, with the left eye at nine feet, with the right at 12 feet. After the discontinuance of the hypodermic injections, on the 11th of October, 1873, fingers were counted with the left at 8 feet, and with the right at 8 to 9 feet. On the other hand, no effect upon the visual field was apparent.

I observed the same effect in two other patients. However, I do not entertain any special hope for the therapeutics of this disease, since the improvement attained by this means remains stationary after it attains a certain degree. Still these effects of strychnia were, to me, most unexpected and interesting.

---

Antidote to Strychnia.—The *Jour. of Applied Chem.* says it is asserted that salad of oil, promptly applied, is an antidote to strychnia. The remedy has not been tried on men, but on dogs; a half pint of oil is said to be sufficient to prevent fatal results.

